# Supplementary material for: Dynamic remodelling of the human host cell proteome and phosphoproteome upon enterovirus infection
Source: Nat Commun. 2020 Aug 28;11:4332. doi: 10.1038/s41467-020-18168-3 (PMC7455705; doi:10.1038/s41467-020-18168-3)
Supplement: Supplementary file 1 — Supplementary Information [file 41467_2020_18168_MOESM1_ESM.pdf]

SUPPLEMENTARY INFORMATION

Dynamic remodelling of the human host cell proteome and phosphoproteome upon enterovirus infection

Giansanti et al.

SUPPLEMENTARY TEXT

Phosphorylation of host factors involved in formation of viral replication organelles

We observed an early increase / late decrease in phosphorylation (cluster 2) of GBF1 T1337 (Supplementary Figure 8b and Source Data File). GBF1 T1337 phosphorylation leads to Golgi disassembly[2,3] and the timing of T1337 phosphorylation increase coincides with Golgi disassembly during CVB3 replication, hinting to a possible connection. T1337 is phosphorylated by 5'-AMP-activated protein kinase (AMPK)[2]. We observed early increased (cluster 2) phosphorylation of activating phosphorylation events on T183 on the AMPKα1 isoform and the corresponding residue T172 on AMPKα2, and a reduced phosphorylation (cluster 3) of the inhibitory site S496 on AMPKα1 (Supplementary Figure 8a). Collectively, these changes imply an early increase in AMPK activity following viral infection, which could explain the observed pattern of GBF1 T1337 phosphorylation. Strikingly, AMPK-dependent GBF1 T1337 phosphorylation is also induced during infection with another positive-strand RNA virus, hepatitis C virus, and has been proposed to contribute to the formation of the membranous web (which is the name used for hepatitis C virus replication organelles)[4]. We investigated the importance of GBF1 T1337 phosphorylation for infection using a previously established assay[5] that involves disruption of endogenous GBF1 function by treatment with the GBF1 inhibitor brefeldin A (BFA). In this experimental setup, BFA-resistant GBF1 (either the A795E or the M832L variant) is overexpressed, cells are subsequently transfected with a subgenomic viral replicon RNA and treated with BFA. BFA efficiently inhibited CVB3 replication, which could be rescued by a BFA-resistant GBF1 A795E (here denoted wildtype to indicate the absence of mutations in phosphosites) (Supplementary Figure 8c and Source Data File) in line with previous observations[5]. Both the non-phosphorylatable mutant T1337A as well as the phosphomimetic mutant T1337E restored replication (Supplementary Figures 8c and 8d). Together these data suggest that (de)phosphorylation of T1337 is not essential for the function of GBF1 in replication. This finding is in agreement with the recent observation that the GBF1 region downstream of its catalytic sec7 domain is dispensable for virus replication[6].

PI4KB S266 phosphorylation (reduced late in infection, cluster 3, Supplementary Figure 8b and Source Data File), in combination with S258 phosphorylation (not detected), has been tentatively linked to Golgi integrity in one study[7]. Several mutants (T>A, T>E and T>D) of these sites, alone or in combination, supported replication in a previously-established assay using PI4KB knockout cells[8]

(Supplementary Figure 8e and Source Data File), suggesting that S258/S266 phosphorylation is not important for the function of PI4KB in infection. However, fundamental knowledge about the PI4KB S258/S266 phosphosites is lacking and other explanations may be possible, including the option that a currently unknown cooperative effect with other sites exists that was not tested here. Single mutation of the other phosphosites that we detected in our analysis (S277, S428 and S511) nor of the well-studied regulatory site S294 that is phosphorylated by PKD to activate PI4KB[9,10] also did not impair the ability of PI4KB to rescue CVB3 replication (Supplementary Figure 8e and Source Data File).

Finally, we observed a late increase (cluster 1) in OSBP S240 phosphorylation (Supplementary Figure 8b), which has been linked to Golgi dissociation[11]. OSBP S240 is phosphorylated by protein kinase D (PKD)[11]. We detected an increased phosphorylation of two known activating sites on PKD1 and/or PKD3 (the peptides could not be unambiguously ascribed to a specific isoform), while no known regulatory sites on PKD2 were identified. The strongest increase in S240 phosphorylation occurred between 4 and 6 h.p.i, which is also the period around which the Golgi falls apart and replication organelles are formed. Unfortunately, we could not study the importance of OSBP phosphorylation as OSBP knock-out cells are not viable and hence a suitable experimental system is not available.

SUPPLEMENTARY METHODS

Immunoblotting

After the incubation time medium was aspirated, cells were carefully washed with ice-cold PBS and lysed in the plate for 15 min on ice in lysis buffer (50mM Tris-HCl pH7.5, 150mM NaCl, 1% Nonidet P40) supplemented with Complete EDTA-free protease inhibitor cocktail (Roche) and PhosStop phosphatase inhibitor mix (Roche). Cells were scraped with a rubber policeman and transferred to 1.5ml vials on ice and lysis was continued for another 15 min. Cell debris was pelleted by centrifugation for 10 min at 4°C at full speed in a tabletop centrifuge. Supernatants were transferred to clean vials and stored at -20°C.

Lysates were mixed with Laemmli sample buffer and heated 5 min at 95°C to denature proteins. Denatured samples were stored at -20°C. Samples were analysed by SDS-PAGE gel electrophoresis and transferred to nitrocellulose membranes by wet-blotting. Membranes were blocked with 2% BSA in TBS-T (TBS (Tris-buffered saline; 10mM Tris pH7.5, 150mM NaCl) with 0.1% Tween-20) for 1 h at room temperature and incubated overnight at 4°C with primary antibodies diluted in 2% BSA in TBS-T. Blots were washed three times with TBS-T, incubated with infrared dye-conjugated secondary antibodies diluted in 2% BSA in TBS-T for 1 h at room temperature, washed three times with TBS-T and rinsed in TBS and then PBS. Blots were scanned using a Li-Cor Odyssey Fc near-infrared fluorescence imaging system.

For the immunoblotting of LC3 and GAPDH the following alterations were made to the protocol. Cells were detached using trypsin, washed in PBS and resuspended in RIPA buffer (40 mM Tris-HCl pH8, 150 mM NaCl, 0.5% sodium deoxycholate, 1% Triton X-100, 0.1% sodium dodecyl sulphate) supplemented with protease inhibitor cocktail (Roche). Lysis was performed for 30 min on ice and cell fragments were removed by centrifugation at 15,000x*g* for 15 min. Protein concentration was determined by Pierce BCA assay kit (ThermoScientific, Waltham, MA) according to the manufacturer’s instructions. Cell lysates (7.5-10 µg) and positive controls for LC3I and LC3II (Nanotools #1041/PC3/LC3I and #1042/PC3/LC3II) were subjected to SDS-PAGE gel electrophoresis. Membranes were probed as described above with the exception that 0.25% fish skin gelatin (FSG, Sigma-Aldrich) in PBS-T was used as blocking buffer. Blots were washed five times in blocking buffer prior to secondary labelling using an antibody conjugated to horseradish peroxidase (HRP) and washed five times in PBS-T and three times in PBS prior to imaging. Blots were incubated with ECL solution (SuperSignal West

Dura Extended Duration Substrate, ThermoScientific) and imaged using a Bio-Rad ChemiDoc imaging system and the accompanying Image Lab software (Bio-Rad).

Antibodies

Primary monoclonal antibodies raised in rabbits were against p-4EBP1(T70) (Cell Signaling #9455, 1:1,000), p-4EBP1(S65) (Cell Signaling #9451, 1:1,000), p-4EBP1(T37/46) (Cell Signaling #2855, 1:1,000), non-p-4EBP1(T46) (Cell Signaling #4923, 1:1,000), 4EBP1 (Cell Signaling #9644, 1:1,000), p-RPS6(S240/244) (Cell Signaling #5362, 1:1,000), p-eEF2(T56) (Cell Signaling #2331, 1:1,000), eEF2 (Cell Signaling #2332, 1:1,000). Polyclonal rabbit antibodies were against CVB3 2C[12] (obtained from Lindsay Whitton, 1:1,000), EMCV capsid (obtained from Ann Palmenberg, 1:1,000). Primary monoclonal antibodies raised in mouse were against RPS6 (Santa Cruz sc-74459, 1:1,000), enterovirus VP1 clone 5D8/1 (Dako # M706401-2, 1:1,000), LAMP1 (Biolegend # 328602, 1:1,000), β-actin (Sigma, 1:30,000), tubulin (Sigma #T9026, 1:2,000), LC3 (Nanotools clone 5F10, 1:500), and GAPDH (Abcam Ab9484, 1:2,000). Secondary goat-anti-rabbit-IRDye800CW and goat-anti-mouse-IRDye680RD were from (Li-Cor, 1:5,000-1:10,000). Secondary goat-anti-mouse-HRP was from (Jackson ImmunoResearch Labaratories, 1:10,000).

Quantitative reverse-transcriptase PCR (qRT-PCR)

At the indicated time points, medium was aspirated and cells were lysed in lysis buffer + 1% tris(2-carboxyethyl)phosphine (TCEP). In some experiments, cell lysates were stored at -20°C. RNA was isolated using the NucleoSpin mini RNA isolation kit (Macherey-Nagel) according to the manufacturer's instructions and mRNA concentrations were checked using the Nanodrop. cDNA was generated using the Taqman Reverse Transcription Reagents (Applied Biosystems) with random hexamer primers according to the manufacturer's instructions. qRT-PCR was performed using the LightCycler 480 SYBR Green I master mix (Roche) for 45 cycles (10 sec at 95°C, 5 sec at 58°C, and 30s at 72°C) in a LightCycler 480 (Roche). Primers against lysosomal target genes (TFEB, LAMP1, TPP1, SCPEP1, GNS, BLOC1S1 and BLOC1S3) are described in[1], against actin in[13] and against CVB3 viral RNA in[14]. Fold change of expression of a gene compared to first time point and normalised to actin in the series was calculated according to the ΔΔCt method.

Replication rescue experiments

HeLa cells grown in a 96-well plate were transfected with plasmids for the expression of GBF1 phosphorylation mutants in the context of a BFA-resistant A795E mutation in the Sec7 domain[5]. The next day the cells were transfected with the CVB3 replicon RNA with the *Renilla* luciferase gene replacing the capsid coding region. After replicon RNA transfection the cells were placed in the medium containing the cell permeable *Renilla* luciferase substrate EnduRen (Promega) and the indicated amount of BFA. Luciferase signal was recorded from live cells every hour for 18 hours after replicon transfection. Wild-type (WT) and PI4KB-knockout ($PI4KB^{KO}$) HeLa cells grown in 96-well plates were transfected with plasmids for the expression of PI4KB (wild-type or mutants)[8]. As controls, kinase-dead (KD) PI4KB or GFP targeted to the Golgi through the transmembrane domain of galactosyltransferase (GalT) were used. The next day, cells were infected with RLuc-CVB3 virus at MOI 0.1 for 8 hrs cells were lysed and luciferase activity was determined using the *Renilla* luciferase assay (Promega).

SUPPLEMENTARY REFERENCES

1. Sardiello, M. *et al.* A gene network regulating lysosomal biogenesis and function. *Science* **325**, 473–477 (2009).
2. Miyamoto, T. *et al.* AMP-activated protein kinase phosphorylates Golgi-specific brefeldin A resistance factor 1 at Thr1337 to induce disassembly of Golgi apparatus. *J. Biol. Chem.* **283**, 4430–4438 (2008).
3. Mao, L. *et al.* AMPK phosphorylates GBF1 for mitotic Golgi disassembly. *J. Cell Sci.* **126**, 1498–1505 (2013).
4. Hansen, M. D. *et al.* Hepatitis C virus triggers Golgi fragmentation and autophagy through the immunity-related GTPase M. *Proc. Natl. Acad. Sci. U. S. A.* **114**, E3462–E3471 (2017).
5. Lanke, K. H. *et al.* GBF1, a guanine nucleotide exchange factor for Arf, is crucial for coxsackievirus B3 RNA replication. *J. Virol.* **83**, 11940–11949 (2009).
6. Viktorova, E. G. *et al.* A Redundant Mechanism of Recruitment Underlies the Remarkable Plasticity of the Requirement of Poliovirus Replication for the Cellular ArfGEF GBF1. *J. Virol.* **93**, e00856-19 (2019).
7. Heilmeyer L. M., J., Vereb G., J., Vereb, G., Kakuk, A. & Szivak, I. Mammalian phosphatidylinositol 4-kinases. *IUBMB Life* **55**, 59–65 (2003).
8. Lyoo, H. *et al.* ACBD3 is an essential pan-enterovirus host factor that mediates the interaction between viral 3A protein and cellular protein PI4KB. *MBio* **10**, (2019).
9. Hausser, A. *et al.* Protein kinase D regulates vesicular transport by phosphorylating and activating phosphatidylinositol-4 kinase IIIbeta at the Golgi complex. *Nat. Cell Biol.* **7**, 880–6 (2005).
10. Hausser, A. *et al.* Phospho-specific binding of 14-3-3 proteins to phosphatidylinositol 4-kinase III beta protects from dephosphorylation and stabilizes lipid kinase activity. *J. Cell Sci.* **119**, 3613–21 (2006).
11. Nhek, S. *et al.* Regulation of oxysterol-binding protein Golgi localization through protein kinase D-mediated phosphorylation. *Mol. Biol. Cell* **21**, 2327–2337 (2010).
12. Cornell, C. T., Kiosses, W. B., Harkins, S. & Whitton, J. L. Inhibition of protein trafficking by Coxsackievirus B3: multiple viral proteins target a single organelle. *J. Virol.* **80**, 6637–6647 (2006).
13. Feng, Q. *et al.* MDA5 detects the double-stranded RNA replicative form in picornavirus-infected cells. *Cell Rep.* **2**, 1187–1196 (2012).
14. Melia, C. E. *et al.* Escaping host factor PI4KB inhibition: Enterovirus genomic RNA replication in the absence of replication organelles. *Cell Rep.* **21**, 587–599 (2017).
15. Schwanhäusser, B. *et al.* Global quantification of mammalian gene expression control. *Nature* **473**, 337–342 (2011).
16. McPhail, J. A. *et al.* Characterization of the c10orf76- PI4KB complex and its necessity for Golgi PI4P levels and enterovirus replication. *EMBO Rep.* **21**, e48441 (2020).

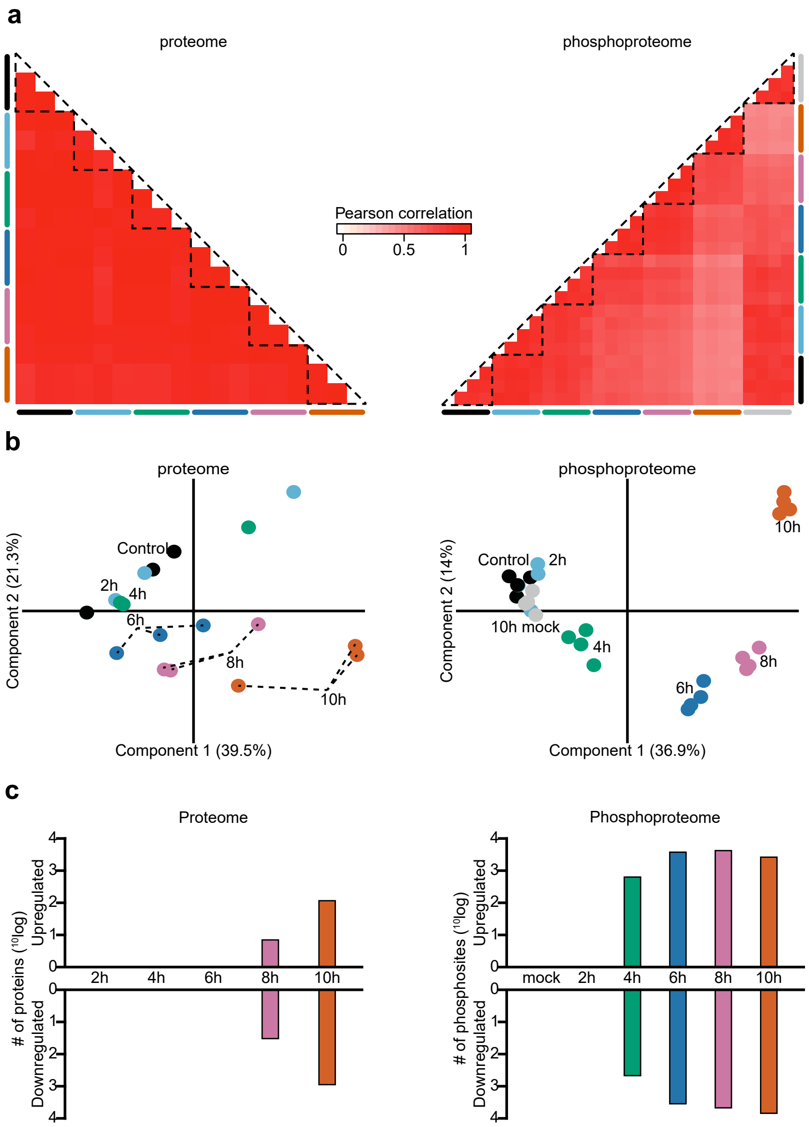

Supplementary Figure 1. Overview of changes in (phospho)proteome during infection.
a. Heat map of Pearson correlation coefficients showing high reproducibility in protein (top) and phosphosite (bottom) LFQ intensity between biological replicates. **b.** Principal component analysis (PCA) of proteome (left) and phosphoproteome (right) samples. Infected samples from 6 h (proteome) or 4 h (phosphoproteome) onward become increasingly distinct. **c.** Number of proteins (left) or phosphosites (right) that are changed at each time point compared to the 0 h mock infected control. Large-scale phosphorylation changes occur already at 4 h post infection (hpi), precede changes in protein levels and that phosphorylation changes are far more extensive than protein level changes. While decreased protein levels are more prevalent than increased protein levels, upregulated and downregulated phosphorylation events are of similar prevalence. Source data are provided as a Source Data file for **c**, **d**, and **e**.

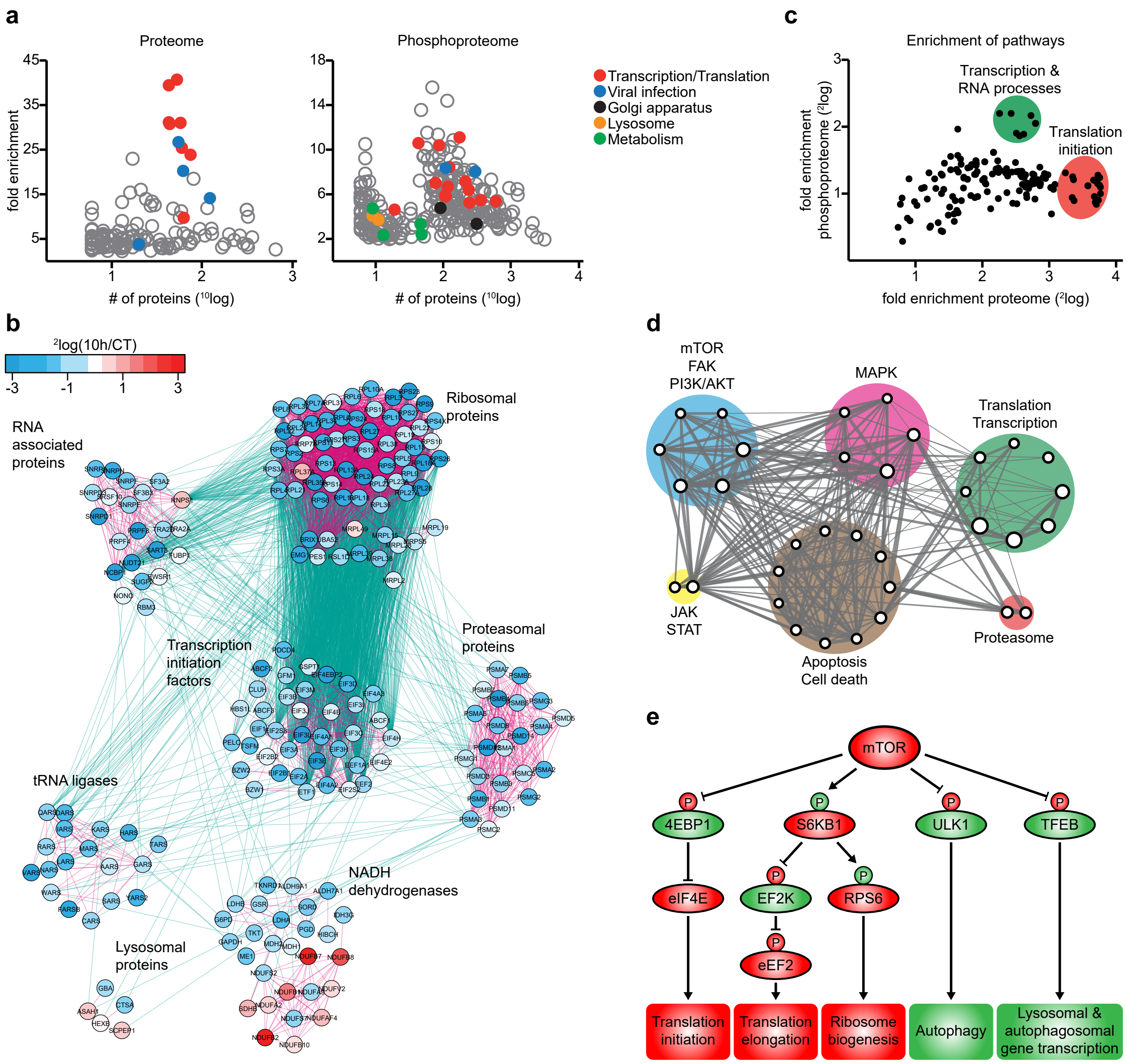

Supplementary Figure 2. Gene ontology and network analysis.

a. GO term analysis of regulated proteins and phosphosites, based upon Supplementary Data 4. Significantly enriched terms were calculated in R with the topGO package. **b.** Sub-networks of proteins down- and up-regulated at 10 hpi. Network was obtained from BioGRID and STRING databases by querying only the statistically significant hits (two-sided Welch t-test, FDR < 0.05) and their interactions using "high confidence" threshold (score > 0.7, STRING). Only STRING experimental, neighbourhood, and database lines of evidence were used. Proteins are color-coded according to the fold change 10 hpi -vs- control untreated cells. Interactions among proteins in the same functional cluster are coloured in magenta, whereas interactions among proteins of different clusters are represented in green. **c.** Statistically significant enriched Reactome pathways (Fisher's exact test) when using the regulated proteins or phosphoproteins as input. d. Signalling networks analysis of the CVB3-regulated proteins and phosphosites (Supplementary Data 2). Each white dot represents a distinct pathway identified by PhosphoPath, using the Wikipathways database as reference. Pathways are manually grouped into six main categories according to biological function/signalling network they are involved in. The size of a dot reflects the number of proteins involved in that pathway, while the width of the gray lines indicates the number of proteins that are shared between the different signalling nodes. **e.** Simplified schematic overview of the mTORC1 signalling cascade and the activity of proteins in the pathway in enterovirus infected cells. Green ovals indicate activation of a protein in CVB3 infected cells, red indicates inhibition. Phosphorylations are indicated with 'P' in circles; green indicates activating phosphorylation events, while red indicates inhibitory phosphorylations. Green rectangles indicate activated cellular processes in infected cells, while red indicates reduced activity. Source data are provided as a Source Data file for **a**, **b**, **c**, and **d**.

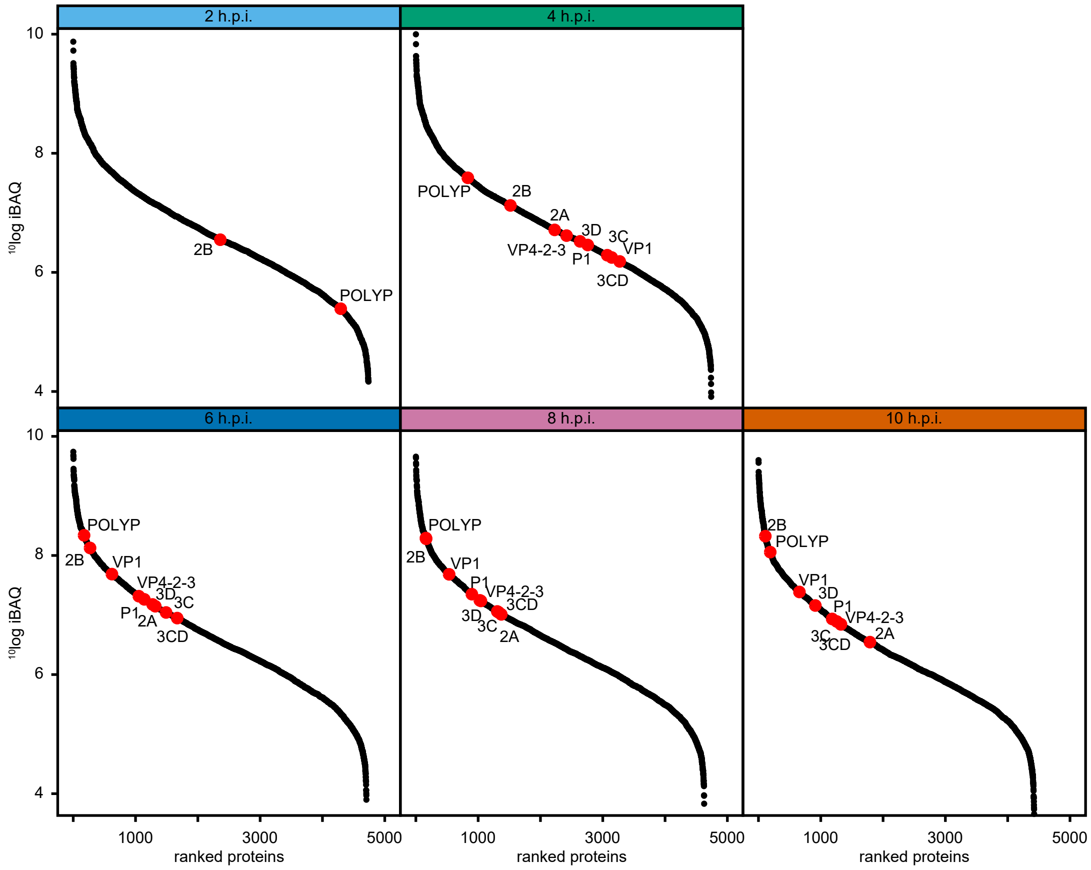

Supplementary Figure 3. Viral protein abundance in the proteome dataset.
Ranked protein abundances from the highest to the lowest. The average iBAQ[15] value across the biological triplicates is shown. During infection, viral protein levels increase dramatically in line with the high levels of viral protein production. Because the viral proteins are derived from a polyprotein that is stepwise proteolytically processed, some peptides produced upon the LysC/trypsin digestion cannot be unambiguously assigned to a precursor or mature viral protein. Therefore, the least processed form to which the detected peptide is unique is indicated (e.g. VP4-2-3 or POLYP [polyprotein]). Source data are provided as a Source Data file.

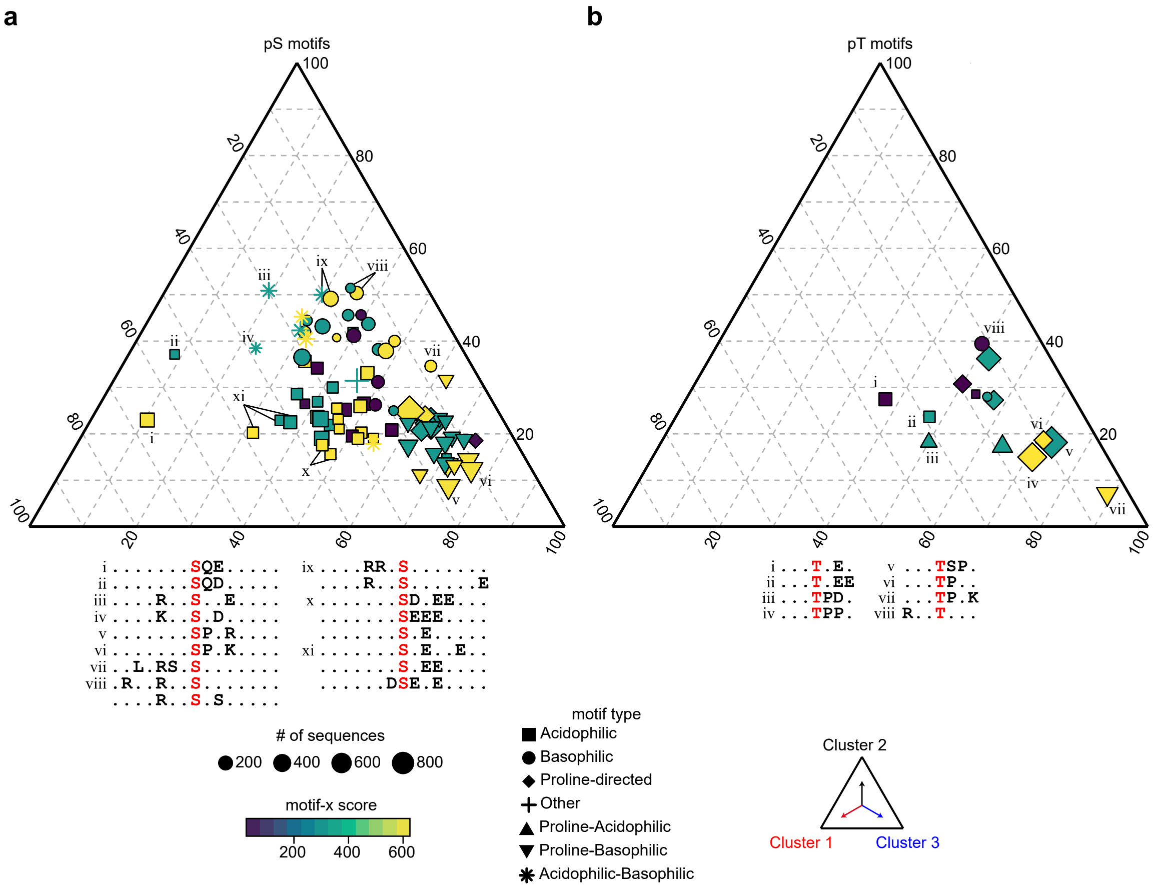

Supplementary Figure 4. Motif-x analysis of phosphorylated sites.

a. Ternary plot of the linear phosphoserine kinase motifs enriched in the tree clusters according to motif-x. Colours are based on the score calculated by motif-x and sizes are based on the numbers of phosphosites matching each motif. Several representative motifs are displayed and their position in the plot is highlighted. **b.** Ternary plot of the linear phosphothreonine kinase motifs enriched in the three clusters according to motif-x, displayed as in panel **a**. Source data are provided as a Source Data file for all panels.

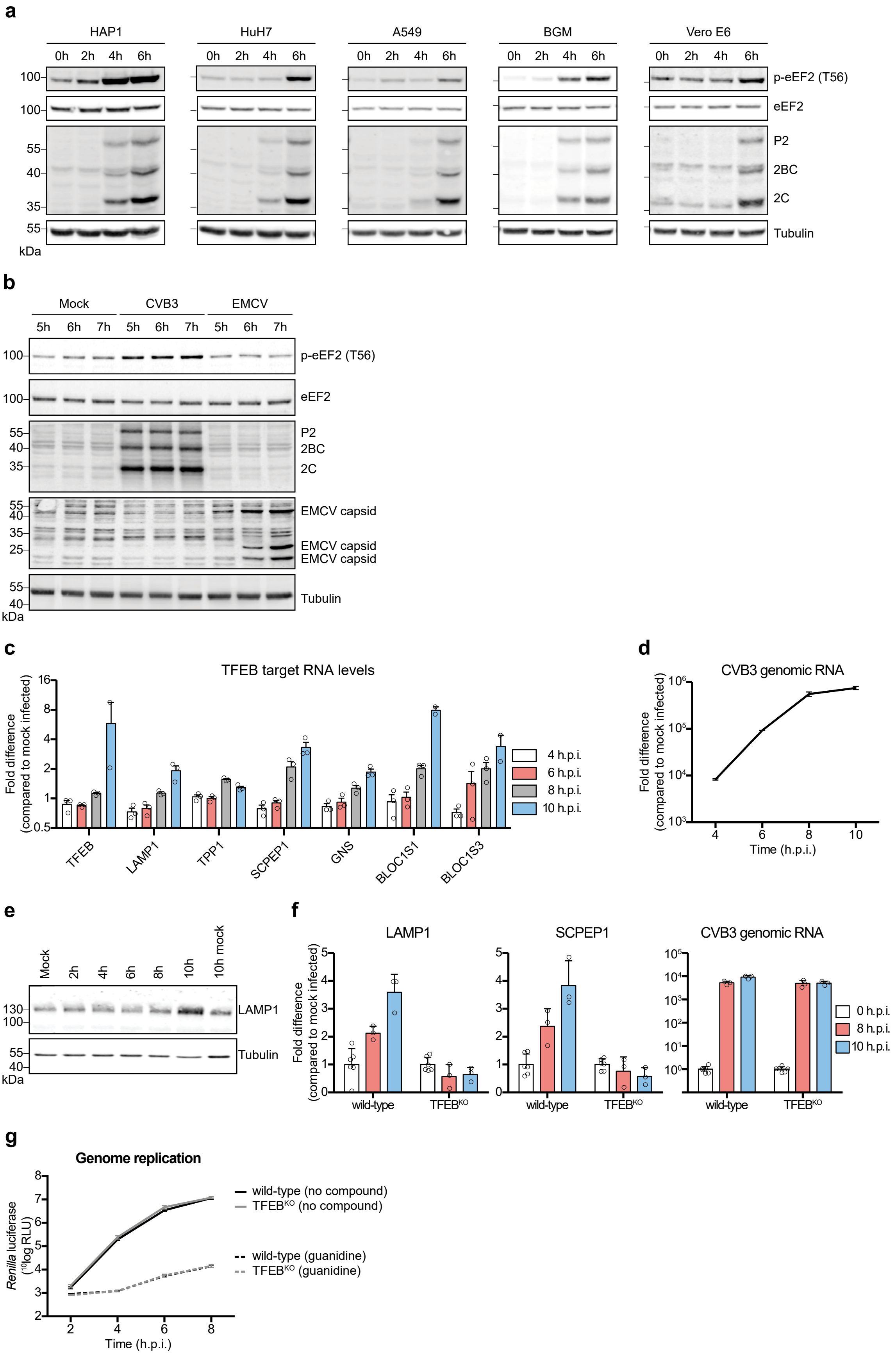
a
HAP1
HuH7
A549
BGM
Vero E6
0h 2h 4h 6h
p-eEF2 (T56)
eEF2
P2
2BC
2C
Tubulin
100
55
40
35
kDa
b
Mock
CVB3
EMCV
5h 6h 7h
p-eEF2 (T56)
eEF2
P2
2BC
2C
EMCV capsid
Tubulin
25
c
TFEB target RNA levels
Fold difference (compared to mock infected)
4 h.p.i.
6 h.p.i.
8 h.p.i.
10 h.p.i.
TFEB
LAMP1
TPP1
SCPEP1
GNS
BLOC1S1
BLOC1S3
d
CVB3 genomic RNA
Time (h.p.i.)
e
Mock
2h
4h
6h
8h
10h
10h mock
LAMP1
130
f
LAMP1
SCPEP1
CVB3 genomic RNA
wild-type
TFEB^KO
0 h.p.i.
8 h.p.i.
10 h.p.i.
g
Genome replication
Renilla luciferase (^10log RLU)
wild-type (no compound)
TFEB^KO (no compound)
wild-type (guanidine)
TFEB^KO (guanidine)
Time (h.p.i.)

Supplementary Figure 5. Inhibition of mTORC1 and activation of TFEB during CVB3 infection.
a. Phosphorylation of eEF2 T56 is induced by CVB3 regardless of the cellular background. Different cell lines (human HuH7, A549 and HAP1 cells; monkey BGM and Vero E6 cells) were infected with CVB3 at MOI 10. Cells were lysed at indicated time points and lysates were analysed by Western blotting as in Figure 4a. **b**. CVB3 induces eEF2 T56 phosphorylation, but the cardiovirus EMCV does not. HeLa cells were infected with either CVB3 or EMCV at MOI 15, cells were lysed at different time points and lysates were analysed by Western blotting as in Figure 4a. Mengovirus capsid, which recognises multiple cleavage forms, serves as an infection control for EMCV. **c.** TFEB targets are upregulated upon CVB3 infection. HeLa cells were infected with CVB3 at MOI 50 as in Figure 4a. At different time points, cells were lysed, RNA was extracted, cDNA was made and the expression levels of a set of genes that are known to be under control of TFEB were determined by qRT-PCR. In parallel, viral genome levels were determined as a control for infection (see panel d). Shown are means, error bars represent ± SEM (n=3 independent experiments, each with three biological replicates). A plot showing the individual data points is available in the Source data file. **d**. Increase of CVB3 genomic RNA levels analysed by qRT-PCR from the same samples as in panel c serves as an infection control. Shown are means, error bars represent ± SEM (n=3 independent experiments, each with three biological replicates) A plot showing the individual data points is available in the Source data file. **e.** The TFEB target protein LAMP1 is increased in infected cells. Lysates of CVB3-infected cells from Figure 4a were analysed by Western blot using an antibody against lysosome-associated membrane protein 1 (LAMP1). **f.** Upregulation of lysosomal genes upon CVB3 infection depends on TFEB. $HAP1^{WT}$ and $TFEB^{KO}$ cells were infected with CVB3 at MOI 10, cells were lysed at the indicated time points and RNA levels were determined by qRT-PCR as in panel c (mRNA levels of LAMP1 and SCPEP1) or panel d (CVB3 genomic RNA levels). Shown are means, error bars represent ± SD (n=3 independent experiments, each with three biological replicates). Plots showing the individual data points are available in the Source data file. **g.** CVB3 replication is not affected by TFEB knockout. $HAP1^{WT}$ and $TFEB^{KO}$ cells were infected with *Renilla* luciferase-expressing CVB3 reporter virus at MOI 0.01 as described in Figure 1a. At the indicated time points cells were lysed and luciferase levels were determined as a quantitative measure of genome replication. The replication inhibitor guanidine was included as a control that shows that infection and translation levels were comparable between cell lines. Shown are means, error bars represent ± SEM (n=3 independent experiments, each with three biological replicates). A plot showing the individual data points is available in the Source data file. Source data are provided as a Source Data file for all panels.

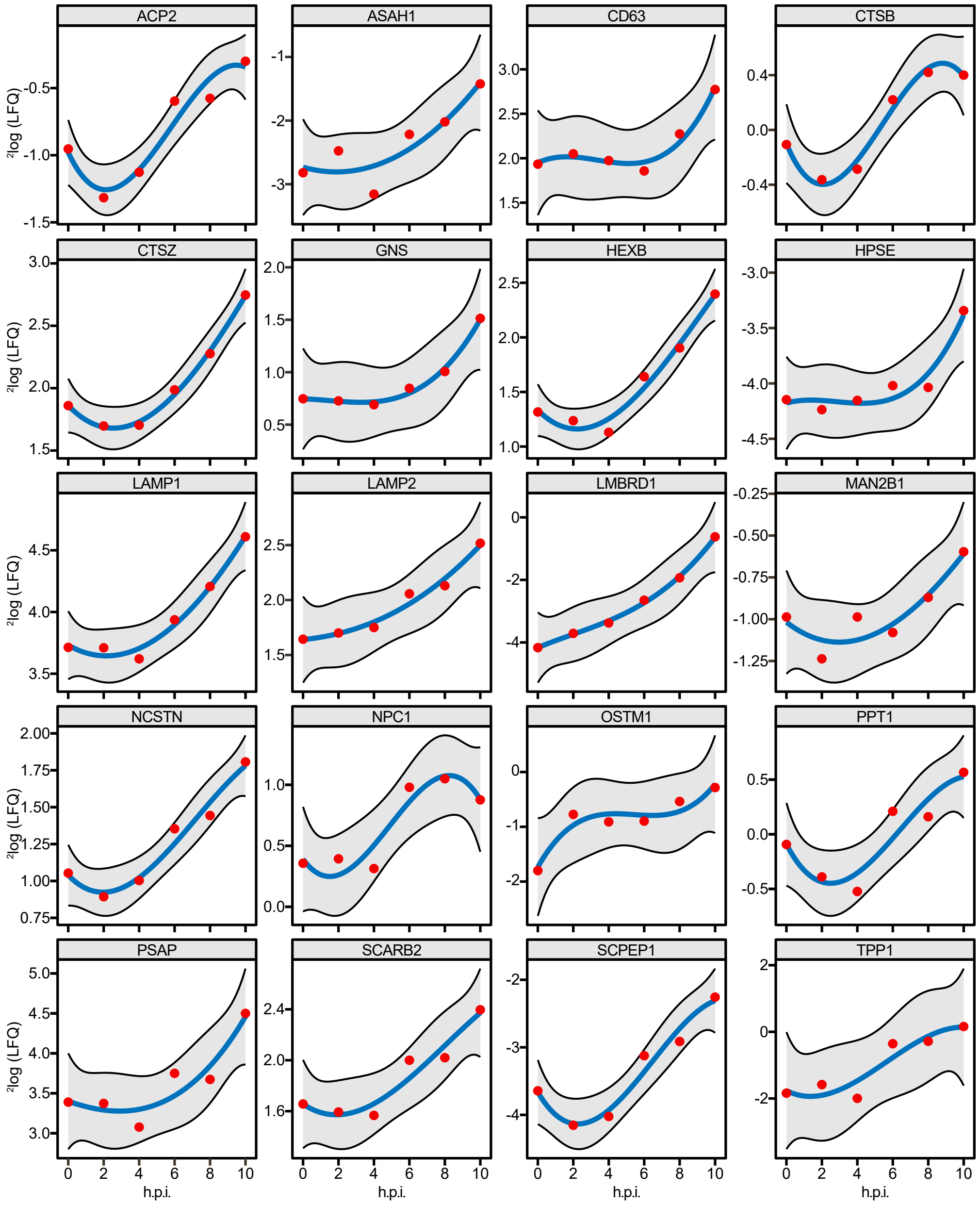

Supplementary Figure 6. Levels of lysosomal proteins during CVB3 infection.
Profile plots of identified and quantified lysosomal proteins during CVB3 infection. Red points are the mean value of the normalized LFQ intensities from the 3 replicates, filled grey area corresponds to 95 % confidence interval, while the blue line is the fitted regression curve. Source data are provided as a Source Data file.

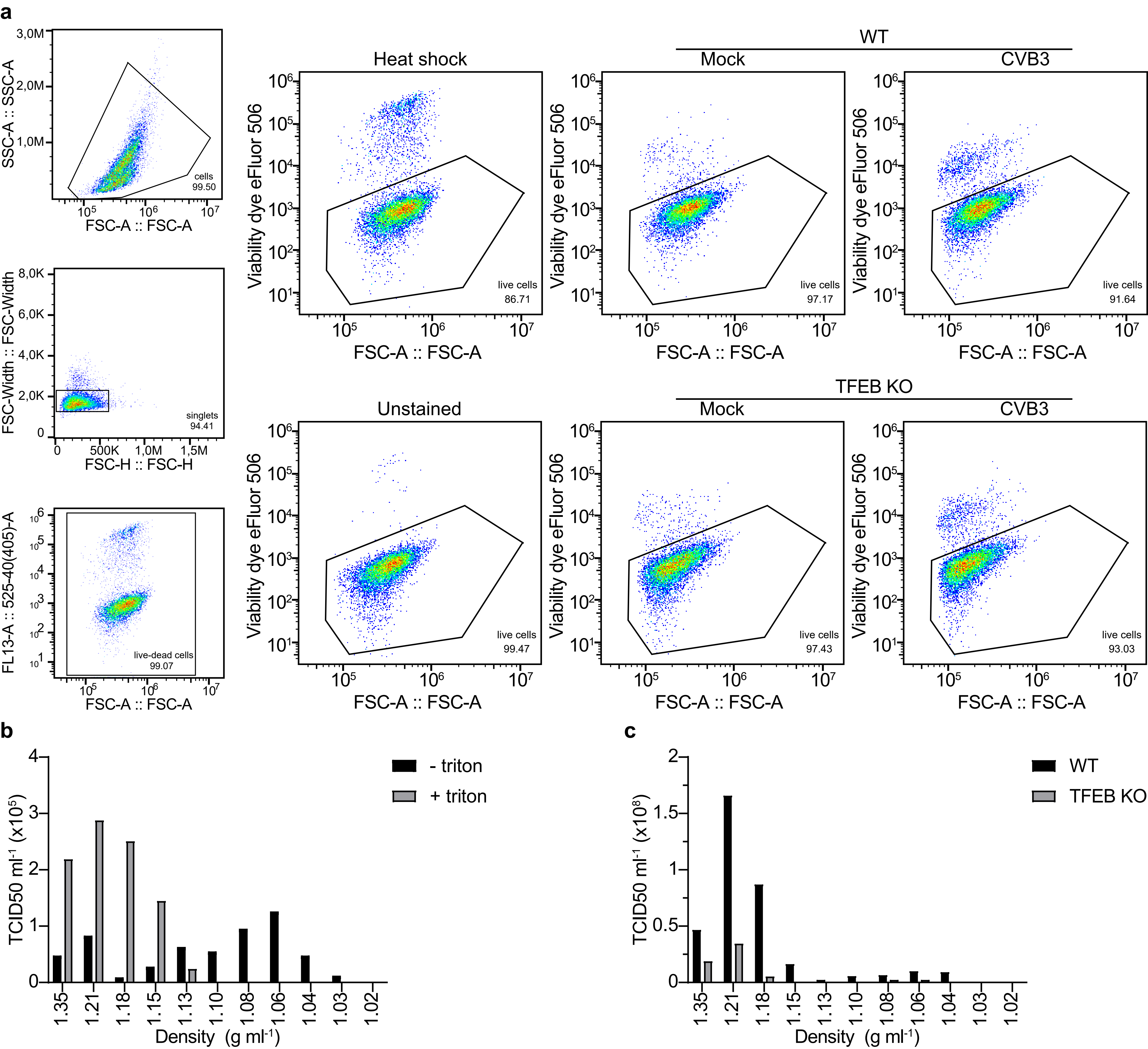
a
SSC-A :: SSC-A
FSC-A :: FSC-A
cells
99.50
FSC-Width :: FSC-Width
FSC-H :: FSC-H
singlets
94.41
FL13-A :: 525-40(405)-A
live-dead cells
99.07
WT
TFEB KO
Heat shock
Unstained
Mock
CVB3
Viability dye eFluor 506
live cells
86.71
97.17
91.64
99.47
97.43
93.03
b
- triton
+ triton
TCID50 ml-1 (x10^5)
Density (g ml-1)
1.35
1.21
1.18
1.15
1.13
1.10
1.08
1.06
1.04
1.03
1.02
c
WT
TFEB KO
TCID50 ml-1 (x10^8)
Density (g ml-1)

Supplementary Figure 7. Assessing non-lytic virus release in CVB3-infected cells.
a. Cell integrity upon CVB3 infection of HAP1^{WT} of TFEB^{KO} HAP1 cells. Gating strategy and representative pseudocolored density plots of three independent experiments for the viability assay of Figure 5b. Heat-shocked cells were included as positive controls in all experiments. **b.** Separation of naked and EV-enclosed CVB3 based on buoyant density. 100,000x*g* pelleted material from CVB3 infected cells was treated with 0.1% Triton-X100 to disrupt any membranes or left untreated before floatation into a density gradient. A shift of the infectivity from low-density fractions (1.10-1.04 g ml^{-1}) to higher density fractions (1.35-1.15 g ml^{-1}) upon detergent treatment is observed, indicating that infectious virus present in low-density fractions is enclosed in extracellular vesicles. **c.** Representative plot from three independent experiments showing the infectivity in different density gradients fractions for HAP1^{WT} and TFEB^{KO} HAP1 cells. Virus was at isolated from cell culture supernatants 8 hpi by pelleting at 100,000x*g* and floated into a density gradient. Source data are provided as a Source Data file for panels **b** and **c**.

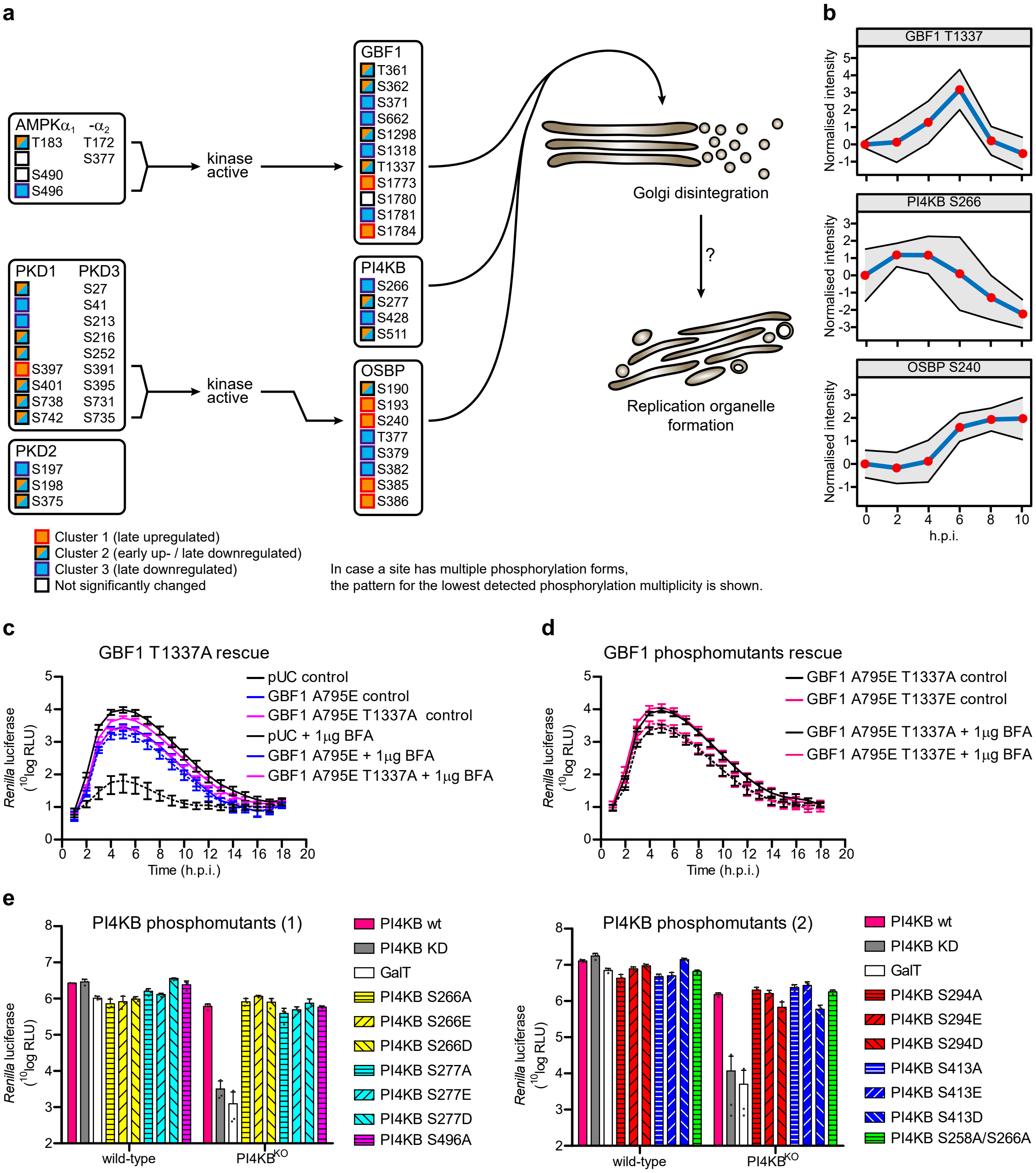

a
AMPKα1 -α2
T183 T172
S377
S490
S496
kinase active
PKD1 PKD3
S27
S41
S213
S216
S252
S397 S391
S401 S395
S738 S731
S742 S735
PKD2
S197
S198
S375
kinase active
GBF1
T361
S362
S371
S662
S1298
S1318
T1337
S1773
S1780
S1781
S1784
PI4KB
S266
S277
S428
S511
OSBP
S190
S193
S240
T377
S379
S382
S385
S386
Golgi disintegration
?
Replication organelle formation
Cluster 1 (late upregulated)
Cluster 2 (early up- / late downregulated)
Cluster 3 (late downregulated)
Not significantly changed
In case a site has multiple phosphorylation forms,
the pattern for the lowest detected phosphorylation multiplicity is shown.
b
GBF1 T1337
PI4KB S266
OSBP S240
Normalised intensity
h.p.i.
c
GBF1 T1337A rescue
pUC control
GBF1 A795E control
GBF1 A795E T1337A control
pUC + 1μg BFA
GBF1 A795E + 1μg BFA
GBF1 A795E T1337A + 1μg BFA
Renilla luciferase (10log RLU)
Time (h.p.i.)
d
GBF1 phosphomutants rescue
GBF1 A795E T1337A control
GBF1 A795E T1337E control
GBF1 A795E T1337A + 1μg BFA
GBF1 A795E T1337E + 1μg BFA
Renilla luciferase (10log RLU)
Time (h.p.i.)
e
PI4KB phosphomutants (1)
PI4KB wt
PI4KB KD
GalT
PI4KB S266A
PI4KB S266E
PI4KB S266D
PI4KB S277A
PI4KB S277E
PI4KB S277D
PI4KB S496A
Renilla luciferase (10log RLU)
wild-type
PI4KB KO
PI4KB phosphomutants (2)
PI4KB wt
PI4KB KD
GalT
PI4KB S294A
PI4KB S294E
PI4KB S294D
PI4KB S413A
PI4KB S413E
PI4KB S413D
PI4KB S258A/S266A
Renilla luciferase (10log RLU)
wild-type
PI4KB KO

Supplementary Figure 8. Phosphorylation of host factors involved in enterovirus replication organelle formation.

a. Analysis of phosphosites on host factors known to be involved in enterovirus replication organelle formation and some upstream kinases, based upon the data in Supplementary Data 4. For a detailed description, see the supplementary text. **b.** Normalised phosphorylation of selected phosphosites on host factors that are linked to Golgi structure, based upon the data in Supplementary Data 4. Red points are the mean values of the normalised phosphosite intensities from the 4 replicates, filled grey area corresponds to 95 % confidence interval, and the blue line is the fitted regression curve. **c.** GBF1 A795E expression restores CVB3 replication upon BFA treatment. HeLa cells transiently expressing the BFA-resistant GBF1 mutant A795E (with or without the T1337A mutation) were transfected with CVB3 replicon RNA containing a *Renilla* luciferase reporter gene and incubated in the presence or absence of 1 μg ml^{-1} of BFA. *Renilla* luciferase signal was recorded from live cells every hour. Empty vector pUC was used as a negative control. Shown are means, error bars represent ± SD (n=2 independent experiments, each with 16 biological replicates). **d.** Non-phosphorylatable T1337A and phosphomymetic T13317E GBF1 mutants similarly rescue CVB3 replicon replication from BFA inhibition. The experiment was performed as in panel c with phosphosite mutants in the background of the BFA-resistant A795E mutant. Shown are means, error bars represent ± SD (n=2 independent experiments, each with 16 biological replicates). **e.** PI4KB phosphomutants similarly rescue RLuc-CVB3 replication in $PI4KB^{KO}$ cells. Wild-type HeLa cells (WT) and $PI4KB^{KO}$ cells transiently expressing PI4KB mutants were infected with RLuc-CVB3, cells were lysed and the *Renilla* luciferase signal was determined. As negative controls Golgi-targeted GFP with the membrane anchor of galactosyltransferase (GalT) and kinase-dead PI4KB (PI4KB KD) were used. For S277 and S428 we studied the S>A, S>D and S>E mutants as specific mutants of these sites have not been described to our knowledge. For S511 we used only the previously described non-phosphorylatable S>A mutant but not the S>D and S>E mutants as these do not faithfully mimic the phosphorylated state of S511 (which is S496 in most common isoform)[16]. Additionally, we studied mutants the well-known regulatory site S294 of which the non-phosphorylatable S>A mutant has been previously described[9,10]. Shown are means, error bars represent ± SD (n=3 independent experiments, each with three biological replicates). Plots showing the individual data points are available in the Source data file. Source data are provided as a Source Data file for **b**, **c**, **d**, and **e**.
