# Supplementary material for: Dynamic remodelling of the human host cell proteome and phosphoproteome upon enterovirus infection
Source: Nat Commun. 2020 Aug 28;11:4332. doi: 10.1038/s41467-020-18168-3 (PMC7455705; doi:10.1038/s41467-020-18168-3)
Supplement: Supplementary file 3 — Description of Additional Supplementary Files [file 41467_2020_18168_MOESM3_ESM.docx]

**Description of Supplementary Files**

**File Name: Supplementary Data 1**

**Description:** Proteome dataset

**File Name: Supplementary Data 2**

**Description:** GO and PhosphoPath analysis of the proteome and phosphoproteome datasets

**File Name: Supplementary Data 3**

**Description:** CVB3 peptides and proteins identifications

**File Name: Supplementary Data 4**

**Description:** Phosphoproteome dataset

**File Name: Supplementary Data 5**

**Description:** Motif-x enrichment analysis and NetworKIN upstream kinase predictions
