# Supplementary material for: Dynamic remodelling of the human host cell proteome and phosphoproteome upon enterovirus infection
Source: Nat Commun. 2020 Aug 28;11:4332. doi: 10.1038/s41467-020-18168-3 (PMC7455705; doi:10.1038/s41467-020-18168-3)
Supplement: Supplementary file 9 — Reporting Summary [file 41467_2020_18168_MOESM9_ESM.pdf]

nature research

Corresponding author(s): Frank van Kuppeveld
Last updated by author(s): Aug 4, 2020

# Reporting Summary

Nature Research wishes to improve the reproducibility of the work that we publish. This form provides structure for consistency and transparency in reporting. For further information on Nature Research policies, see our Editorial Policies and the Editorial Policy Checklist.

## Statistics

For all statistical analyses, confirm that the following items are present in the figure legend, table legend, main text, or Methods section.

| n/a | Confirmed | |
|---|---|---|
| ☐ | ☒ | The exact sample size (*n*) for each experimental group/condition, given as a discrete number and unit of measurement |
| ☐ | ☒ | A statement on whether measurements were taken from distinct samples or whether the same sample was measured repeatedly |
| ☐ | ☒ | The statistical test(s) used AND whether they are one- or two-sided<br>*Only common tests should be described solely by name; describe more complex techniques in the Methods section.* |
| ☒ | ☐ | A description of all covariates tested |
| ☐ | ☒ | A description of any assumptions or corrections, such as tests of normality and adjustment for multiple comparisons |
| ☐ | ☒ | A full description of the statistical parameters including central tendency (e.g. means) or other basic estimates (e.g. regression coefficient) AND variation (e.g. standard deviation) or associated estimates of uncertainty (e.g. confidence intervals) |
| ☐ | ☒ | For null hypothesis testing, the test statistic (e.g. *F*, *t*, *r*) with confidence intervals, effect sizes, degrees of freedom and *P* value noted<br>*Give P values as exact values whenever suitable.* |
| ☒ | ☐ | For Bayesian analysis, information on the choice of priors and Markov chain Monte Carlo settings |
| ☒ | ☐ | For hierarchical and complex designs, identification of the appropriate level for tests and full reporting of outcomes |
| ☐ | ☒ | Estimates of effect sizes (e.g. Cohen's *d*, Pearson's *r*), indicating how they were calculated |

*Our web collection on statistics for biologists contains articles on many of the points above.*

## Software and code

Policy information about availability of computer code

| | |
|---|---|
| Data collection | Mass spectrometry data was collected using commercial software (Xcalibur v3.0.63, Thermo Scientific) associated with the specific mass spectrometer for each experiment. |
| Data analysis | 1. Mass spectrometry raw files were processed with MaxQuant (1.5.8.0), using the embedded MaxLFQ algorithm.<br>2. The MaxQuant output results were further processed in Perseus (1.5.3.2), RStudio (1.1.456 and 1.2.5019), and Excel Microsoft Excel 2013.<br>3. Plots were generated with Perseus or RStudio.<br>4. Protein signaling networks were generated in Cytoscape (3.1) via PhosphoPath (3.2).<br>5. GO analysis was performed by topGo (2.40.0).<br>6. Phosphorylation motif analysis was performed with rmotifx (1.0).<br>7. Upstream kinse prediction was performed by NetworKIN (3.0)<br>8. Flow cytometry data were analyzied with FlowJo (10.07)<br>9. The GraphPad Prism (5 and 7) was used to plot the graphs.<br>10. All figures were finally organized in Adobe Illustrator CS6. |

For manuscripts utilizing custom algorithms or software that are central to the research but not yet described in published literature, software must be made available to editors and reviewers. We strongly encourage code deposition in a community repository (e.g. GitHub). See the Nature Research guidelines for submitting code & software for further information.

## Data

Policy information about availability of data

All manuscripts must include a data availability statement. This statement should provide the following information, where applicable:

- Accession codes, unique identifiers, or web links for publicly available datasets
- A list of figures that have associated raw data
- A description of any restrictions on data availability

The mass spectrometry proteomics data have been deposited in the ProteomeXchange Consortium via the PRIDE partner repository with the dataset identifier PXD011163. Annotation from UniprotKB, Gene Onthology (GO), and Kyoto Encyclopedia of Genes and Genomes (KEGG) where downloaded within Perseus. Reactome pathways were obtained by PANTHER (http://pantherdb.org). PhosphoSitePlus, Biogrid, and Wikipathways databases are embedded or directly queried by Cytoscape/PhosphoPath.

# Field-specific reporting

Please select the one below that is the best fit for your research. If you are not sure, read the appropriate sections before making your selection.

☒ Life sciences ☐ Behavioural & social sciences ☐ Ecological, evolutionary & environmental sciences

For a reference copy of the document with all sections, see nature.com/documents/nr-reporting-summary-flat.pdf

# Life sciences study design

All studies must disclose on these points even when the disclosure is negative.

| | |
|---|---|
| Sample size | Statistical methods were not used to predetermine sample size. The sample size (proteome n = 3, phosphoproteome n = 4) was chosen so that the sample variance could be estimated (which requires n >= 2). Due to the higher dynamics of the phosphoproteomes a fourth biological replica was included into the analysis. |
| Data exclusions | No data were excluded from the analysis. |
| Replication | All attempts at replication were successful.<br>Figures 1-3: Experiments were performed once. For the full proteome MS experiment, three biological replicates were analyzed. For the phosphoproteome MS experiment, four biological replicates were analyzed.<br>Figure 4a: Two independent experiments with different cell numbers seeded. For both experiments, cells were subconfluent upon infection.<br>Figure 4b: Two independent experiments.<br>Figure 4c: Performed once.<br>Figure 5a: Three independent experiment, each consisting of biological triplicates.<br>Figures 5b-e: Three independent experiments with single samples per condition.<br>Supplementary Figures 1-4: Same experiment as Figure 1.<br>Supplementary Figure 5a: Two independent experiments.<br>Supplementary Figure 5b: Performed once.<br>Supplementary Figure 5c-d: Three independent experiments with biological triplicates.<br>Supplementary Figure 5e: Same experiment as Figure 4a.<br>Supplementary Figure 5f-g: Three independent experiments with biological triplicates.<br>Supplementary Figure 6: Same experiment as Figure 1 (full proteome).<br>Supplementary Figure 7a: Three independent experiments.<br>Supplementary Figure 7b: Performed once.<br>Supplementary Figure 7c: Three independent experiments.<br>Supplementary Figure 8a-b: Same experiment as Figure 1.<br>Supplementary Figure 8c-d: Experiments were performed at least twice with 16 biological replicates per experiment.<br>Supplementary Figure 8e: Three independent experiments. |
| Randomization | No randomization was applied in our sample sets given the small number of samples and the lack of influence of randomization on the experimental design and experimental approach used in the study. However, cell culture work and mass spectrometry analysis were performed by different researchers. |
| Blinding | This was not a blinded study, but cell culture and mass spectrometry analysis were performed by different researchers. For data acquisition and data analysis, blinding was not necessary or advised (in-vitro experiments required prior knowledge for data interpretation) to reduce any bias of the statistical analysis. |

# Reporting for specific materials, systems and methods

We require information from authors about some types of materials, experimental systems and methods used in many studies. Here, indicate whether each material, system or method listed is relevant to your study. If you are not sure if a list item applies to your research, read the appropriate section before selecting a response.

## Materials & experimental systems

| n/a | Involved in the study |
| --- | --- |
| ☐ | ☒ Antibodies |
| ☐ | ☒ Eukaryotic cell lines |
| ☒ | ☐ Palaeontology and archaeology |
| ☒ | ☐ Animals and other organisms |
| ☒ | ☐ Human research participants |
| ☒ | ☐ Clinical data |
| ☒ | ☐ Dual use research of concern |

## Methods

| n/a | Involved in the study |
| --- | --- |
| ☒ | ☐ ChIP-seq |
| ☐ | ☒ Flow cytometry |
| ☒ | ☐ MRI-based neuroimaging |

# Antibodies

Antibodies used

1. Rabbit mAb anti-p-4EBP1(T70) (Cell Signaling #9455, 1:1,000)
2. Rabbit mAb anti-p-4EBP1(S65) (Cell Signaling #9451, 1:1,000)
3. Rabbit mAb anti-p-4EBP1(T37/46) (Cell Signaling #2855, 1:1,000)
4. Rabbit mAb anti-non-p-4EBP1(T46) (Cell Signaling #4923, 1:1,000)
5. Rabbit mAb anti-4EBP1 (Cell Signaling #9644, 1:1,000)
6. Rabbit mAb anti-p-RPS6(S240/244) (Cell Signaling #5362, 1:1,000)
7. Rabbit mAb anti-p-eEF2(T56) (Cell Signaling #2331, 1:1,000)
8. Rabbit mAb anti-eEF2 (Cell Signaling #2332, 1:1,000)
9. Rabbit pAb anti-CVB3 2C (not commercially available, obtained from Lindsay Whitton, 1:1,000)
10. Rabbit pAb anti-EMCV capsid (not commercially available , obtained from Ann Palmenberg, 1:1,000)
11. Mouse mAb anti-RPS6 (Santa Cruz sc-74459, 1:1,000)
12. Mouse mAb anti-enterovirus VP1 clone 5D8/1 (Dako # M706401-2, 1:1,000)
13. Mouse mAb anti-LAMP1 (Biolegend #328602, 1:1,000)
14. Mouse mAb anti-β-actin (Sigma #A5316, 1:30,000)
15. Mouse mAb anti-tubulin (Sigma #T9026, 1:2,000)
16. Mouse mAb anti-LC3 (Nanotools #0231-100/LC3-5F10 clone 5F10, 1:500)
17. Mouse mAb anti-GAPDH (Abcam Ab9484, 1:2,000)
18. Goat-anti-rabbit-IRDye800CW (Li-Cor #926-32211, 1:5,000-1:10,000)
19. Goat-anti-mouse-IRDye680RD (Li-Cor #926-68070, 1:5,000-1:10,000)
20. Goat-anti-mouse-HRP(Jackson ImmunoResearch Laboratories # 115-035-003, 1:10,000)

Validation

Commercial antibodies were validated by the suppliers, see the supplier's websites. In particular Cell Signaling adopts six complementary strategies to determine the validity of an antibody (see https://www.cellsignal.com/contents/our-approach/cst-antibody-validation-principles/ourapproach-validation-principles). For the other suppliers, the exact validation process is unknown to the investigators. For validation of the LC3 antibody, a commercially available cell lysate enriched for LC3I and LC3II (Nanotools, catalogue #1041/PC3/LCeI and #1043/PC3/LC3II) was taken along as positive control in all experiments (Figure 5d, e). Banding pattern was consistent with that previously reported for the different modification forms of LC3 (Mizushima N., Yoshimori T. How to interpret LC3 immunoblotting. Autophagy 3, 542-5, (2007)). For validation of the anti-CVB3 2C antibody, see reference 74. Furthermore, as control uninfected cells were taken along in the same experiment. For the anti-EMCV capsid antibody uninfected cells were taken along in the same experiment. No additional validation was performed for the anti-EMCV antibody.

# Eukaryotic cell lines

Policy information about cell lines

Cell line source(s)

1. HeLa R19 cells were obtained from George Belov (University of Maryland, College Park (MD), USA). Cells are not commercially available.
2. BGM cells were obtained from ATCC.
3. Vero E6 cells were obtained from ATCC.
4. HuH7 cells were obtained from ATCC.
5. A549 cells were obtained from the National Institute of Public Health and the Environment (RIVM), Bilthoven, The Netherlands. Commercially available via ATCC.
6. HAPl (wt and TFEB-KO) were from Horizon Discoveries.

Authentication

The cell lines derived from ATCC or Horizon Discoveries were authenticated by the suppliers. None of the cell lines used were authenticated by the investigators.

Mycoplasma contamination

We confirm that all cells were tested as mycoplasma negative.

Commonly misidentified lines
(See ICLAC register)

No commonly misidentified cell lines were used.

# Flow Cytometry

## Plots

Confirm that:

☒ The axis labels state the marker and fluorochrome used (e.g. CD4-FITC).

☒ The axis scales are clearly visible. Include numbers along axes only for bottom left plot of group (a 'group' is an analysis of identical markers).

☒ All plots are contour plots with outliers or pseudocolor plots.

☒ A numerical value for number of cells or percentage (with statistics) is provided.

## Methodology

| | |
|---|---|
| Sample preparation | Cells were harvested from tissue culture flasks at the indicated time points post infection. Detached cells present in the cell culture supernatant were isolated by pelleting at 200xg. Adherent cells belonging to the same samples were washed in PBS, trypsinized and resuspended in medium. The collected cells were pooled, washed twice in cold PBS and stained for 30 min on ice using Fixable Viability Dye eFluor 506 (eBioscience, San Diego, CA) according to the manufacturer's protocol. As negative control and positive control, unstained cells and heat shocked cells were taken along respectively. To generate heat shocked cells, cells were incubated at 65 °C for three min, followed by immediate placement on ice for one min. Heat-killed cells were mixed 1:1 with untreated cells prior to staining to serve as gating reference. Following staining, unbound dye was removed by washing with PBS and the cells were fixed in 1% paraformaldehyde (PFA). After fixation samples were washed in 1% BSA in PBS and analyzed. |
| Instrument | CytoFLEX LX (Beckman Coulter). |
| Software | FlowJo vl0.07 software (FlowJo, Ashland, OR, USA). |
| Cell population abundance | In the FACS experiment performed a small sampling of the cells was taken to assess cell viability. Analysis of cell viability represents a qualitative assessment of cell state, and is therefore exclusively reported as percentage live/dead cells in literature. In the case of a lytic virus infection, % cell viability (but not absolute cell population abundance) furthermore provides a measure of infection progression and expected virus release of the total virus pool as over the course of infection a transition between no and complete (100%) lysis of infected cells occurs. |
| Gating strategy | Ten thousand events were recorded for each sample. First, a SSC-A:FSC-A gate was used to remove background detected in the buffer solution (<5%). Next, singlets were gated based on a FSC-width:FSC-height gate. Finally, live and dead cells were gated (>7000 cells) based on eFluor 506 fluorescence (525-540):FSC-A, with the cut-off between negative and positive cells set based on the heat shocked control sample (see above and Supplementary Figure 7a). |

☒ Tick this box to confirm that a figure exemplifying the gating strategy is provided in the Supplementary Information.
